# Metasurfaces with Phase-Change Materials for Mid-Wave Infrared Thermal Management

**DOI:** 10.3390/mi17010017

**Published:** 2025-12-24

**Authors:** Viktoriia E. Babicheva, Heungsoo Kim, Alberto Piqué

**Affiliations:** 1 Department of Electrical and Computer Engineering, University of New Mexico, MSC01 1100, 1 University of New Mexico, Albuquerque, NM 87131, USA; 2 Naval Research Laboratory, 4555 Overlook Ave, Washington, DC 20375, USAalberto.pique.civ@us.navy.mil (A.P.)

**Keywords:** optical antennas, vanadium dioxide, thermochromic, insulator-to-metal transition, Planck thermal emission, thermal radiation, engineered emissivity coating

## Abstract

Applying coatings that suppress the radiance changes related to temperature-dependent blackbody emission enables temperature-independent optical and sensing systems. Phase-change materials can significantly modify their optical properties within their transition window, but compensating for the large mid-wave infrared (MWIR, 3–5 µm) variation is demanding: blackbody radiance at 3 µm increases nearly 10-fold as the temperature rises from 30 °C to 80 °C. Vanadium dioxide VO_2_, whose insulator–metal transition offers a sharp contrast and a low-loss insulating state, is attractive for applications in thermal management, but simple thin-film designs cannot provide full compensation. We demonstrate metasurface coatings that provide this compensation by constructing an array of metal–VO_2_–metal antennas tuned to maintain constant thermal emission at a target wavelength over a temperature range of 30 °C to 80 °C. Antennas of several lateral sizes are combined, so their individual resonances collectively track the Planck change. This design provides both optical contrast and the correct temperature derivative, which are unattainable with homogeneous layers. Our approach results in a negligible apparent temperature change of the metasurface across the 30–80 °C range, effectively masking thermal signatures from MWIR detectors stemming from the low losses of VO_2_.

## 1. Introduction

Suppressing thermal emissions to counter infrared imaging using active or passive thermal regulation is an active field of research aimed at concealing individuals or objects from detection [1,2,3,4,5]. These approaches are particularly useful against systems that rely on sensing thermal signatures, and thermal stealth can be achieved using various strategies and materials engineered to reduce or eliminate detectability by infrared sensors [6]. Thermal imaging technologies, such as infrared cameras, can detect the temperature of distant surfaces by capturing their thermal distributions. Concealing thermal data can involve imitating or blending with ambient heat patterns (with structures often referred to as ‘chameleons’), primarily to evade infrared surveillance [2,3,7]. Effective thermal emission control can be achieved through insulation, active cooling, or the use of engineered materials that manage radiative output [8,9]. A key challenge is to design thermal camouflage systems that remain effective under significant temperature changes and in extreme thermal environments [10,11]. The Stefan–Boltzmann law determines thermal emission from a surface at a given temperature, and recent efforts have been directed at offsetting these blackbody changes [12,13,14,15]. Phase-change materials enable efficient, reconfigurable control of photonic response at the nano- and micro-scales [16,17,18]. Recent works have introduced coatings based on phase-change materials that overcome standard temperature-dependent behavior [2,3,19,20]. These efforts focus on developing adaptive coatings with tunable emissivity across wide temperature ranges, with potential applications in thermal control, sensing, energy harvesting, and thermophotovoltaics [21,22,23].

A material’s phase transition profoundly affects its thermal and electronic behavior, thereby influencing its emissivity across different wavelengths. Emitters can be engineered to compensate for changes in blackbody radiation within particular temperature ranges through such phase transitions. Phase-change materials, such as vanadium dioxide VO_2_, rare-earth nickelates, and germanium telluride, show reversible transitions between crystalline phases or between amorphous and crystalline states, causing substantial shifts in their optical characteristics [1,2,3,6,21,24,25,26,27,28,29,30,31,32,33,34,35,36,37]. One notable example is VO_2_, which undergoes a transition from a metallic state at elevated temperatures to an insulating state below ~70 °C. This phase change results in abrupt shifts in both the electrical conductivity and the optical response [38,39,40]. VO_2_ thin films have been extensively studied for modulating thermal emission, with the metallic and insulating phases of VO_2_ enabling adjustable emissivity, and VO_2_ is particularly promising for smart thermal devices due to its pronounced change in infrared reflectance near the transition temperature. Hysteresis in phase-change materials presents a challenge, making it difficult to precisely manage the switching thresholds between the insulating and metallic phases [41,42,43,44]. However, doping VO_2_ with tungsten can soften the transition, offering benefits such as smoother thermal behavior, minimized hysteresis, and enhanced material stability [45,46,47,48,49,50]. The phase-transition temperature of undoped VO_2_ films is ~68 °C and gradually decreases to 50 °C and 35 °C when the W-doping content increases from 1% up to 2%, respectively. As the W-doping content increases, the transition amplitude of VO_2_ films decreases, and the transition occurs in a broad temperature range. Beyond VO_2_, materials such as germanium telluride and indium antimonide telluride have been explored for phase-change–driven electrical and optical modulation [51,52,53,54,55]. Rare-earth nickelates provide tunable transition temperatures via composition [56,57,58], layering [59], structural relaxation [60,61], and proton doping [62]. Samarium–neodymium superlattices enable phase transition control, while samarium nickelate stabilizes infrared output near 120 °C [2,3,32,63,64,65,66].

In the mid-wave infrared (MWIR) range, typically spanning wavelengths 3–5 μm, the design of optical antennas encounters unique limitations that preclude the effective use of traditional plasmonic and dielectric resonant strategies. Metals in this regime behave as near-perfect electrical conductors, exhibiting high negative real permittivity and a negligible penetration depth, which effectively suppresses the formation of well-defined plasmonic resonances. As a result, the MWIR range lies in a challenging intermediate regime where conventional subwavelength resonance mechanisms break down, requiring alternative design approaches. To address these limitations, antennas operating in the MWIR regime must be engineered to rely on geometric or structural resonances, such as Fabry–Pérot-like cavity modes in ultra-thin films or leaky guided modes in planar resonators, whose spectral behavior can be finely tuned by controlling thickness modulation and hybridization with thin-layer modes. Arrays of such antennas, when arranged into metasurfaces, offer a viable route to tailoring the effective spectral emissivity and angular selectivity of the surface [4,5,14,67,68]. The optical response in these metastructures is governed not by the material’s inherent resonance, but by the collective response of the individual elements [69,70,71]. Furthermore, in this regime, the strong metallicity of available materials introduces additional design constraints, such as increased Joule losses and limited mode confinement in metal-based geometries. To mitigate these issues, designers often employ metal–insulator configurations with optimized nanogap spacing to achieve capacitive field enhancement, even in the absence of true plasmonic resonance. These effects can be exploited to create metasurfaces with spectrally selective absorption or emission profiles that align with atmospheric transparency windows or thermal imaging requirements. The engineered coupling of geometric resonances enables the realization of highly functional surfaces for MWIR applications, including tunable thermal emitters, narrowband infrared detectors, and directional radiative control platforms [28,72,73,74,75].

**Figure 1:**
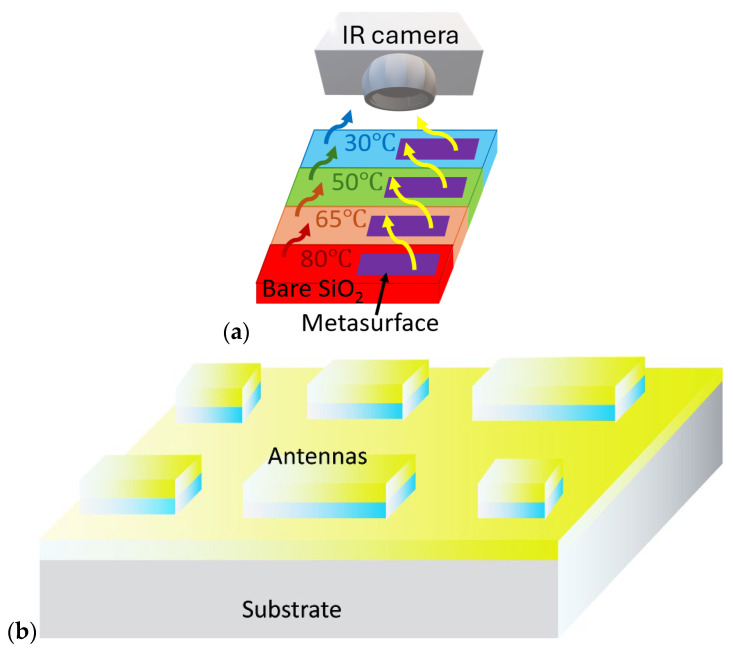
(**a**) Schematic illustration of thermal masking of a heated surface. The unmasked surface increases its thermal radiance upon heating. In contrast, an ideal concealed surface enhanced by a metasurface maintains a consistent low radiance, even when the actual temperature of the surface is increased. (**b**) Proposed metasurface consisting of antennas of different dimensions engineered to cover the required spectral range of 3–5 μm due to the variable spectral position of the resonance of each antenna.

Here, we develop a metasurface that enables active spectral control of thermal emission through temperature-induced phase transitions (Figure 1a). Although compensation in the long-wave infrared (LWIR) range can often be achieved using uniform planar thin films of phase-change materials, extending this approach to the MWIR range is considerably more challenging due to the steep increase in blackbody emission with temperature, especially at shorter wavelengths, such as 3 μm. This work demonstrates that metasurfaces composed of antennas undergoing insulator-to-metal transitions can facilitate coatings with temperature-invariant radiance over the range of 30–80 °C, specifically tailored for the MWIR window of 3–5 μm. The coating’s optical response is engineered to counteract the inherent temperature sensitivity of blackbody radiation, enabling precise control of infrared signatures and stabilized emission characteristics. The metasurface architecture integrates a distribution of antenna elements with varied lateral dimensions (Figure 1b), enabling tunable resonance conditions that collectively generate a compensating spectral response. Integrating such metasurfaces as thermal emitters with near-zero differential radiative output significantly extends the effective operational temperature range. We employ metasurfaces composed of optical antennas engineered to support sharp, tunable resonances that undergo substantial spectral redistribution in response to temperature changes, thereby enabling more effective radiative compensation in the MWIR range. Our results show that combining phase-change materials with resonant nanostructures offers a powerful strategy for achieving thermal coating with controlled and temperature-independent emission in MWIR.

## 2. Experimental

### 2.1. Growth of VO_2_ Films

**Figure 2:**
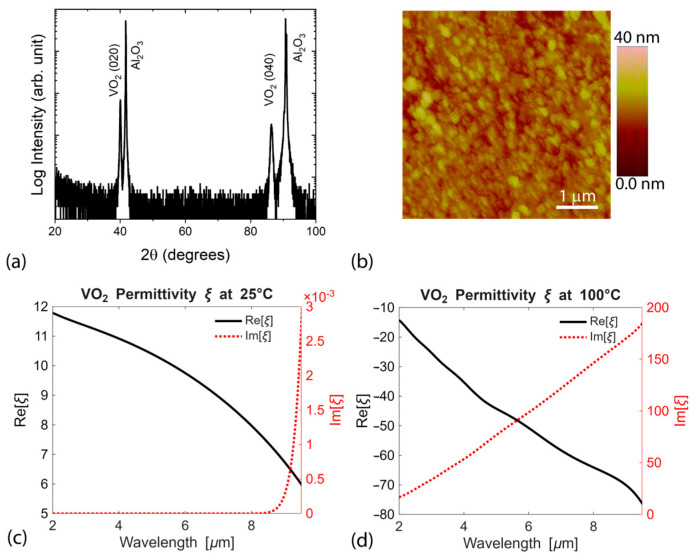
(**a**) XRD 2θ-ω scan of a typical VO_2_ film grown on (0006) Al_2_O_3_ substrate. (**b**) AFM image (5 µm × 5 µm) of VO_2_ film surface showing uniform surface morphology. Optical permittivity of VO_2_ film (**c**) at 25 °C and (**d**) at 100 °C. The VO_2_ film is grown on c-plane Al_2_O_3_ substrates at 550 °C.

In this work, we demonstrate a temperature-independent thermal emitter utilizing a phase change VO_2_ layer, whose insulator-to-metal transition enables tuning of thermal radiation in the MWIR range. VO_2_ films are grown on c-plane sapphire substrates by pulsed laser deposition (PLD). A detailed description of the growth procedure can be found elsewhere (see Refs. [29,76]). Briefly, a KrF excimer laser (Lambda Physik LPX 305, 248 nm, pulse duration 30 ns) operates at a pulse rate of 5 Hz and is focused by a 50-cm focal length lens onto a rotating target at a 45° angle of incidence. The energy density of the laser beam on the target surface is maintained at ~1.5 J/cm^2^ with a target-substrate separation of 5 cm. The VO_2_ target (2.54 cm in diameter) is purchased from American Elements. The deposition chamber is initially pumped down to <1 × 10^−5^ Torr, and during deposition, O_2_ gas is introduced into the chamber to maintain a desired pressure of 20 mTorr. The laser beam is rastered across the target surface in order to uniformly ablate the target surface while the target is rotated. For uniform film depositions, the substrate is also rotated during deposition at a speed of 20 rpm. The substrate temperature during deposition is kept at 550 °C. The samples are cooled down to room temperature at the same oxygen pressure. The crystal structure of the films is characterized by X-ray diffraction (XRD) measurements with Cu Kα1 radiation (Bruker D8). Figure 2a shows a typical XRD pattern of a 65-nm-thick VO_2_ film on an Al_2_O_3_ substrate. The peaks at ~41.68° and ~90.70° are indexed to the planes (0006) and (000 12) of the Al_2_O_3_ substrate, respectively. The peaks at ~39.9° and ~85.9° correspond to M-VO_2_ peaks (M means monoclinic structure) (either 020 and 040 or 002 and 004, respectively). It is observed that films grown at 20 mTorr O_2_ show only M-VO_2_ peaks, indicating that no other phases of VO_2_ are formed during film growth. The surface morphology of VO_2_ is characterized by using an atomic force microscope (AFM Bruker Dimension Icon) operating in peak force mode (Figure 2b). The film is composed of irregularly shaped large grains with a characteristic size of ~100 nm and rounded edges. The film exhibits a relatively low surface roughness of ~2.7 nm.

### 2.2. VO_2_ Permittivity

Figure 2c,d shows the permittivities of a 65-nm VO_2_ film on the sapphire substrate as a function of wavelength at temperatures of 25 °C and 100 °C. At room temperature (~25 °C), VO_2_ exhibits semiconductor-like behavior, characterized by a relatively high real permittivity and low loss, displaying dielectric-like properties. As the temperature increases to ~100 °C, it transitions into a metallic phase, where the real part of permittivity becomes negative and the imaginary part increases significantly, indicating strong absorption. A similar range of permittivity values has been reported for VO_2_ films grown on SiO_2_ and other substrates in earlier works [77,78,79,80,81,82]. These optical constant data are used for simulating the thermal radiation of the hybrid structure comprising VO_2_ and Au antennas.

The optical permittivity values (Re[ξ] and Im[ξ]) of each layer (VO_2_ and Al_2_O_3_) are derived from spectroscopic ellipsometry, where the Re[ξ] represents the real part of the permittivity, and the Im[ξ] represents the imaginary part of the permittivity. Temperature-dependent ellipsometry spectra are measured at wavelengths between 2 µm and 20 µm at incident angles of 55°, 65°, and 75° using a variable angle spectroscopic ellipsometry (IR-VASE, J. A. Woollam) that is equipped with a heating stage [LN2-D2; INSTEC]. The optical permittivity values are then extracted from these spectra by fitting the spectroscopic ellipsometry parameters (Ψ and Δ) using a Drude oscillator model.

The VO_2_ thin films reported in this work serve as a source of experimentally validated material parameters, enabling accurate modeling of the metasurface response across the insulating–metallic transition. The structural and optical characterizations are therefore focused on extracting refractive indices, phase-transition behavior, and film quality necessary for reliable full-wave simulations. We use the optical constants extracted from our own VO_2_ films because the dielectric response is highly dependent on the deposition method and film microstructure. This ensures that the simulated metasurface behavior corresponds to the material quality attainable in our fabrication process. Fabrication and measurement of complete metasurface prototypes fall beyond the scope of the present work but represent an active direction of our ongoing experimental program. These future efforts will directly build on the material data reported here to provide device-level verification of the simulated emissivity modulation.

### 2.3. Antenna Design Rules

The metasurface is introduced as a functional architecture that enables broadband compensation through the collective action of multiple, temperature-responsive resonators. Each unit cell supports a distinct spectral mode arising from its metallic–dielectric–metal configuration and the VO_2_-driven permittivity modulation. As the temperature increases and VO_2_ transitions to its metallic, highly lossy state, the individual resonances of the unit cells are significantly damped due to increased absorption and reduced field confinement within the dielectric spacer. The broadband compensation effect, therefore, arises from how the collective suppression of these resonances reshapes the overall absorption spectrum to offset the intrinsic thermal changes in the unpatterned VO_2_ film. This metasurface-specific mechanism, rooted in the engineered heterogeneity of the resonant profile, is essential for achieving the wideband control.

Because blackbody radiance increases substantially with temperature, the metasurface must alter its emissivity to offset this change to maintain a stable emissivity. The emissivity ratio, therefore, indicates how effectively the structure compensates for the temperature-driven variation in thermal emission. We employ CST Studio Suite to model the electromagnetic response of antennas arranged in periodic arrays. Each antenna is treated as part of an infinite lattice using periodic boundary conditions in the plane, while perfectly matched layers are applied along the propagation axis to emulate open boundaries. The simulations capture both near-field and far-field characteristics, enabling analysis of resonance behavior, scattering, and collective array effects. Material parameters are incorporated as dispersive and lossy, based on experimental data, to ensure realistic predictions. Emissivity is obtained from absorptivity under the assumption of thermal equilibrium, and calculations are carried out across the 3–5 μm mid-infrared spectral range where the metasurface is designed to operate effectively.

**Figure 3:**
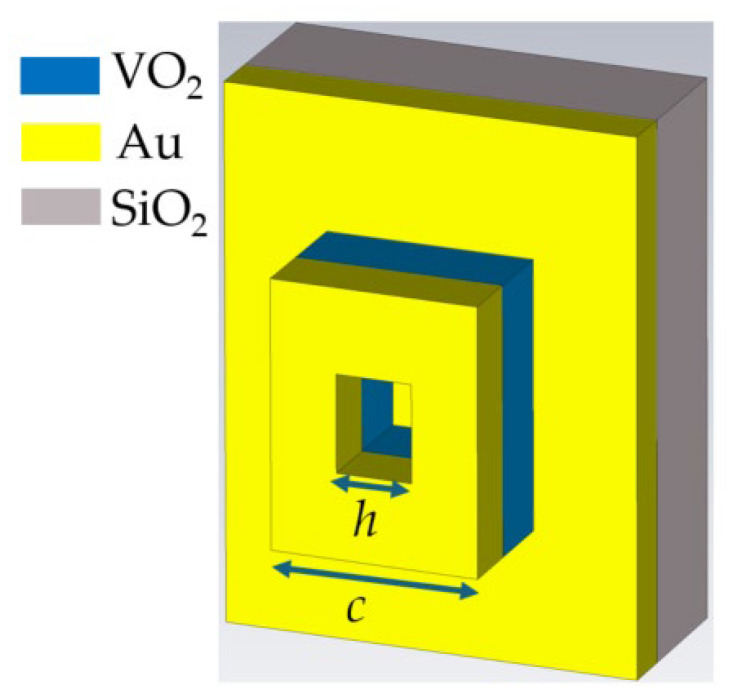
Antennas for control of their emissivity: 3D view of the metasurface with one element (antenna) consisting of a metal-VO_2_-metal stack. The antenna has a square shape, with both sides measuring *c*, and a hole inside with sides *h* in both orthogonal directions. The air hole crosses the upper metal layer and the VO_2_, but not the lower metal layer, and can be straightforwardly incorporated into the lithographic process. The substrate is covered with a continuous 100-nm Au film. VO_2_ and the upper Au layer constitute the antenna with layer thicknesses of 60 nm and 80 nm, respectively.

We perform full-wave numerical simulations and extract emissivity from the reflectance of the metasurface. The model incorporates material dispersion as well as variations due to temperature. In our numerical analysis, we begin by examining a single resonant element composed of a metal–VO_2_–metal stack (Figure 3). Metal–insulator–metal antenna structures are particularly advantageous in the infrared regime, where conventional dielectric antennas lack sufficient mode confinement and traditional plasmonic elements are limited in their effectiveness due to the negligible penetration of light into metal. The structure is built on a substrate uniformly coated with a 100-nm-thick Au film, which serves as the bottom reflective layer. The antennas consist of a 60-nm-thick VO_2_ layer capped with an 80-nm-thick patterned Au layer. The antenna features a lithographically defined square geometry with a central aperture that penetrates through both the upper Au layer and the VO_2_ layer but does not extend into the lower Au film. This configuration enables precise control of the resonant properties and is fully compatible with standard fabrication processes.

In our simulations, emissivity is evaluated through Kirchhoff’s law, which relates the directional emissivity to the absorptance of a structure under thermal equilibrium. Since the proposed multilayer metasurface is designed to be optically opaque in the MWIR spectral range, transmittance is effectively zero, and the absorptance reduces to *A*(λ) = 1 − *R*(λ), where *R*(λ) is the simulated reflectance. The emissivity at each temperature and wavelength is therefore computed as ε(λ,*T*) = 1 − *R*(λ,*T*). The ratios ε(30 °C)/ε(80 °C) are based on these absorptance-derived emissivities, and the compensation performance metrics directly reflect the temperature-induced changes in the MWIR response of the metasurface. Parameters for each metasurface are specified in the Results and Discussion section. Gold and silica data are taken from [83] and [84], respectively.

A practical fabrication route for the proposed metasurface can be outlined using well-established thin-film and lithographic processes. First, a silica (SiO_2_) substrate is coated with a thin gold film deposited by electron-beam evaporation to form the bottom metallic layer. High-resolution patterning of the metasurface features can then be achieved using either electron-beam lithography or advanced optical lithography, followed by development of the resist. After pattern definition, a thin film of VO_2_ is deposited using pulsed-laser deposition or RF sputtering at room temperature, ensuring uniform coverage of the patterned regions. A second gold layer is subsequently deposited on VO_2_/Au/SiO_2_ by electron-beam evaporation to define the top electrodes and complete the multilayer stack. After a lift-off step removes the remaining resist, the sample is annealed at 400 °C for 1 h to achieve high-quality crystalline VO_2_ [85], yielding the patterned Au/VO_2_/Au nanostructures on the silica substrate and demonstrating the practical manufacturability of the proposed design.

## 3. Results and Discussion

### 3.1. Thermal Variation of Radiance and Required Compensation

The total thermal power emitted by an object can be calculated by integrating its spectral radiance over all wavelengths and hemispherical directions. The emitted temperature-dependent spectral radiance that corresponds to any object emitting thermal radiation is I(λ,*T*) = ε(λ)I_BB_(λ,*T*), where λ is the wavelength, *T* is the temperature, I_BB_(λ,*T*) is the blackbody distribution, and ε(λ,*T*) is the spectral emissivity. The total thermal power emitted by a surface is determined by integrating its spectral radiance I(λ,*T*). Even if the spectral emissivity is independent of temperature (that is, ε(λ,*T*) ≈ ε(λ)), the total emissivity ε_tot_(*T*) can still vary slightly with temperature due to the integration of ε(λ) multiplied by the blackbody spectrum I_BB_(λ,*T*). However, this effect is typically minor compared to the dominant *T*^4^ dependence. As a result, the Stefan–Boltzmann law directly links the surface temperature to the emitted power, supporting the familiar concept that hotter objects radiate more energy. This principle underpins technologies such as infrared imaging and non-contact thermometry. However, this direct correlation can be disrupted by utilizing an engineered thermal-emission coating. A perfect cover functions as a constant-power emitter, radiating nearly the same energy regardless of temperature, thereby masking temperature variations from thermal sensors. Such emitters have been termed zero-differential emitters and are categorized by whether they obscure thermal signatures across a broad wavelength range or only at specific spectral bands [2,3,6]. In one example, the power emitted over a selected wavelength range in LWIR remains nearly unchanged across different temperatures [2]. Concealment from hyperspectral cameras and systems with bandpass filters can be achieved using zero-differential spectral emitters, where changes in blackbody output at each wavelength are counterbalanced by corresponding shifts in emissivity [3,6].

**Figure 4:**
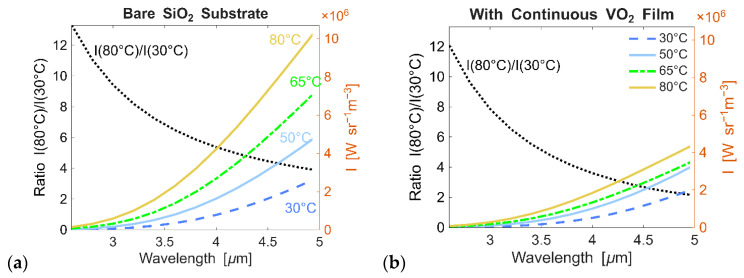
Radiance changes corresponding to different temperatures (30, 50, 65, and 80 °C): (**a**) Bare silica substrate, (**b**) silica substrate with a continuous 200-nm VO_2_ film. The ratios of the radiance values I(80 °C)/I(30 °C) are shown on the left-hand side axes (with I(80 °C)/I(30 °C) = I_BB_(80 °C)/I_BB_(30 °C) because ε(*T*) ≈ const), and the radiance values are on the right-hand side axes. In panel (**a**), the plot shows typical unmasked thermal changes, which are primarily determined by changes in blackbody radiation. In panel (**b**), the ratio decreases only marginally when the VO_2_ film is included, indicating that a continuous VO_2_ film is not sufficient to compensate for changes induced by blackbody radiation.

The typical exposed thermal signature of a hot object is primarily driven by changes in blackbody radiation, and Figure 4a shows the spectral radiance of the substrate at various temperatures. Radiance calculations consider both the blackbody radiation spectrum I_BB_(λ,*T*) and the emissivity of the coating ε(λ), which governs how effectively a material emits thermal energy. The emissivity depends on the intrinsic properties and surface structure of the material, which, in turn, affect both radiation and absorption. Accurate evaluation of radiance, therefore, requires integrating both Planck’s law and material-specific emissivity to capture the actual thermal emission behavior. The SiO_2_ surface emissivity remains constant across the wavelength range because its refractive index changes negligibly with temperature. As predicted by the Stefan–Boltzmann law, higher temperatures result in significantly higher emissions, making the surface appear unmasked.

Thermal coatings designed for operation in LWIR can achieve effective temperature compensation even with uniform planar thin-film structures, primarily due to the relatively modest variation in blackbody radiance across a broad temperature range. For instance, when the temperature increases from 30 °C to 80 °C, the spectral radiance of a blackbody at the wavelength of 8 μm increases by only a factor of approximately 2.3 and at the wavelength of 12 μm by about 1.8. These variations can be counteracted by moderate tuning of the film’s optical properties, provided its material undergoes a phase change. In contrast, at the shorter wavelength of 3 μm, the same temperature change results in a nearly 10-fold increase in radiance (more precisely, it is 9.4), posing a significantly greater challenge for compensation (Figure 4a). In the MWIR range, the spectral shifts achievable through conventional thin-film interference are insufficient to balance the steep increase in emissive power (Figure 4b). To overcome this limitation, alternative design strategies are required. Here, we utilize metasurfaces built from optical antennas designed to exhibit sharp tunable resonances that undergo significant spectral shifts with temperature variations, thereby enhancing radiative compensation within the MWIR range. In the subsequent analysis, we use the ratio I_BB_(80 °C)/I_BB_(30 °C) as a reference for the required compensation and design the antennas and metasurfaces such that the corresponding emissivity ratio transitions in the *opposite* direction ε(30 °C)/ε(80 °C). When the inverse of the emissivity ratio matches the increase in blackbody radiance, the total emitted power remains constant, indicating successful thermal compensation.

### 3.2. Performance of Individual Antennas

**Figure 5:**
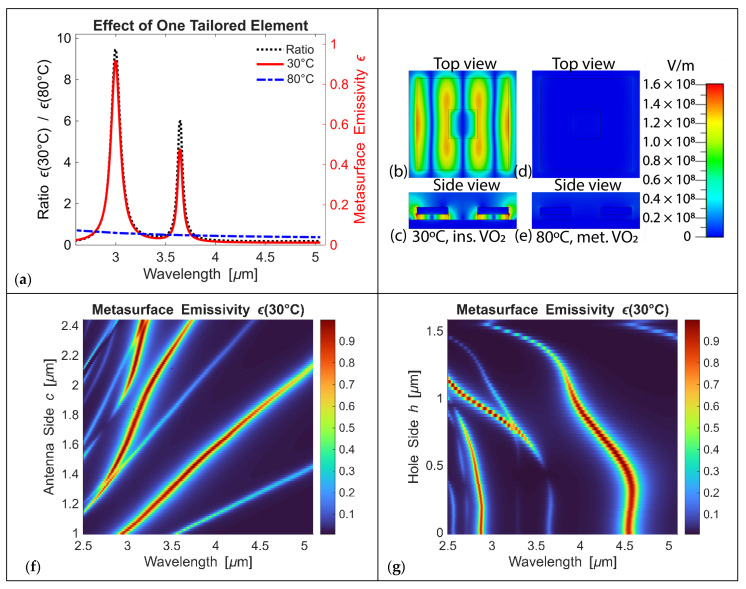
(**a**) Change in metasurface emissivity ε(30 °C)/ε(80 °C) (on the left-hand side axis) and emissivity values ε(30 °C) and ε(80 °C) (on the right-hand side axis) when VO_2_ transitions from the insulator state at the temperature of 30 °C to the metal state at 80 °C. (**b**–**e**) Electric-field distribution (averaged over the period of wave oscillation). (**b**) Top and (**c**) side views for 30 °C, where VO_2_ is insulator, and (**d**) top and (**e**) side views for 80 °C, where VO_2_ is metallic. Top-view cross-sections are taken at the middle of the VO_2_ layer, and side views are taken in the middle of the antenna. Antenna side *c* = 1.025 μm, hole side *h* = 0.325 μm, and the domain side is 1.205 μm (same as in panel (**a**)), and this electric field distribution corresponds to the wavelength of 3 μm. The colormap is the same across all panels. (**f**) Changes in metasurface emissivity as its dimensions vary. The antenna side c varies, while the hole side relates to the antenna side as *h* = *c* − 0.7 μm and the domain size as *c*/0.85. (**g**) Changes in metasurface emissivity as a function of varying hole dimensions.

We compute the variation in metasurface emissivity as VO_2_ undergoes a phase transition from the insulating state at 30 °C to the metallic state at 80 °C. We choose the antenna side *c* = 1.025 μm, the hole side *h* = 0.325 μm, and the domain side is 1.205 μm, and, thus, the antenna geometry is optimized to produce a ~10× change in emissivity at a wavelength of 3 μm (Figure 5a). VO_2_ is well-suited for resonant thermal structures due to its low-loss insulating phase, which supports high-quality resonances with substantial field enhancement within the antenna geometry. Upon transition to the metallic state, VO_2_ exhibits significantly increased optical losses, allowing efficient modulation of the resonance strength and spectral response through a thermally driven phase transition.

To clarify the underlying mechanism responsible for the pronounced emissivity modulation, we include representative electric-field distributions for the VO_2_ insulating and metallic phases (Figure 5b–e). These spatial profiles directly expose how the metasurface supports strongly confined resonant fields at 30 °C, with energy concentrated within and around the patterned VO_2_ layer. When VO_2_ becomes metallic at 80 °C, the same visualizations reveal substantial damping and reduced field penetration, all of which suppress the resonance. Together, the insulating- and metallic-state field maps illustrate how VO_2_-driven changes in confinement and loss give rise to the observed ten-fold emissivity contrast.

Further spectral tuning is achieved by varying the antenna’s lateral dimension (Figure 5f). This parametric variation provides effective control over the resonance response of each metasurface antenna across the operating band, so the overall spectral behavior emerges directly from how the individual elements are shaped, tuned, and arranged relative to one another. We demonstrate a micrometer-scale shift in the peak spectral position in our simulations. Additionally, tuning the hole dimensions independently provides another degree of control over the metasurface emissivity, enabling fine adjustments to the thermal response of the metasurface (Figure 5g). The antenna side is *c* = 1.6 μm, and the domain size is *c*/0.85 = 1.88 μm. It is an effective mechanism for tuning the strength and spectral position of the resonance peak, which spans the entire MWIR range (on the order of microns).

In the metallic state of VO_2_, the antenna response becomes strongly damped, and they have minimal sensitivity to variations in antenna dimensions or hole size. We systematically identify the antenna dimensions required to support resonant excitations within a targeted spectral window, taking into account both geometry and VO_2_ material dispersion. The shifted resonances are obtained by varying antenna geometries, and these resonances form a library of spectrally distinct elements that can be used to construct metasurfaces with customized thermal responses, similarly to what is done for metalenses [86,87]. This dimensional mapping enables the design of a metasurface composed of multiple distinct antenna elements, each contributing a spectrally shifted resonance, collectively spanning a broader range and enhancing the overall compensation performance.

### 3.3. Metasurfaces Consisting of Different Antennas

**Figure 6:**
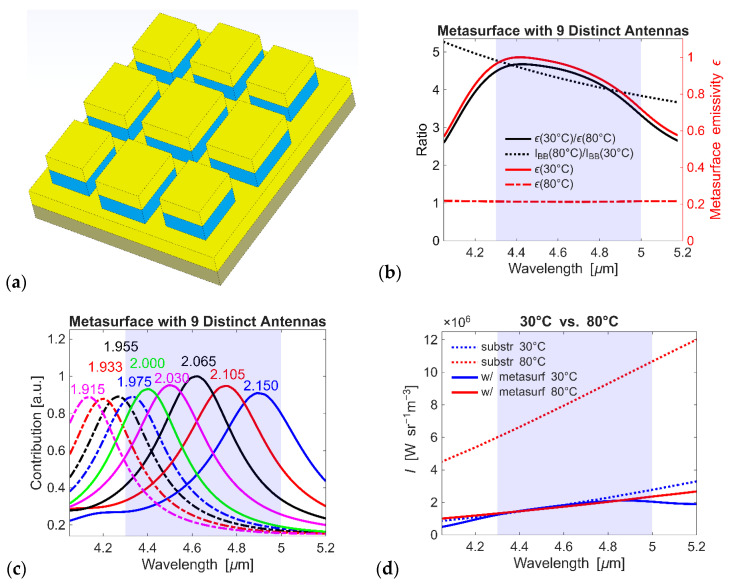
Metasurface design with multiple elements (antennas). (**a**) Schematic of a metasurface with 9 distinct elements. For this design, no holes are required. (**b**) Ratios ε(30 °C)/ε(80 °C) and I_BB_(80 °C)/I_BB_(30 °C) (on the left-hand side axis) and metasurface emissivity values ε(30 °C) and ε(80 °C) (on the right-hand side axis) corresponding to the metasurface with 9 distinct elements (with sides c = 1.915, 1.933, 1.955, 1.975, 2.000, 2.030, 2.065, 2.105, and 2.150 μm). Each metasurface element acts independently and can provide compensation at the required wavelength. The antennas are tailored to compensate for changes in blackbody radiation in the wavelength range λ ≈ 4.3–5.0 μm. The distance between the antennas is 0.8 μm (side-to-side), which is sufficient for negligible interaction between them. (**c**) Contribution of each antenna for the case shown in panel (**a**) is normalized to the maximum value. (**d**) Radiance changes corresponding to temperatures of 30 °C and 80 °C for a bare silica substrate and a metasurface.

Next, we design metasurfaces with multiple antenna elements (Figure 6a). Specifically, the metasurface includes 9 geometrically distinct antennas whose resonances are spectrally close. Our simulations show that antennas with sides *c* = 1.915, 1.933, 1.955, 1.975, 2.000, 2.030, 2.065, 2.105, and 2.150 μm provide the required spectra, and the placement of the distinct 9 elements does not impact the metasurface response as long as the side-to-side distance between the antennas > 0.4 μm. The metasurface is designed to compensate for blackbody changes within the wavelength range λ ≈ 4.3–5.0 μm (Figure 6b). The set of 9 antennas is dimensionally nonuniform, with smaller antennas spaced more closely and larger ones more sparsely, since more substantial compensation is required at shorter wavelengths where smaller antennas operate. We observe that each metasurface element acts independently and can provide compensation at its designated wavelength (Figure 6c). The side-to-side distance between the antennas is fixed at 0.8 μm, which is sufficient to ensure negligible near-field interaction. The average antenna filling factor is approximately 43% of the area, but it can be increased up to 62% without compromising the metasurface performance. Even when the antenna resonances are spectrally close, that is, the closest resonances are ≈0.065 μm (Figure 6c), their peaks remain well-defined, confirming minimal interaction between them. This absence of coupling allows every element, whether its resonance is isolated or overlapping, to function autonomously, enabling metasurfaces assembled from multiple antennas to be accurately tailored for broadband thermal compensation. Spectrally overlapping resonances have Lorentzian shapes, indicating negligible coupling between antennas. The lack of interaction and coupling enables each element to function independently, and the metasurface is constructed from multiple elements. We observe negligible radiance changes that correspond to temperatures of 30 °C and 80 °C for the metasurface, whereas the radiance of the bare substrate changes drastically (Figure 6d).

**Figure 7:**
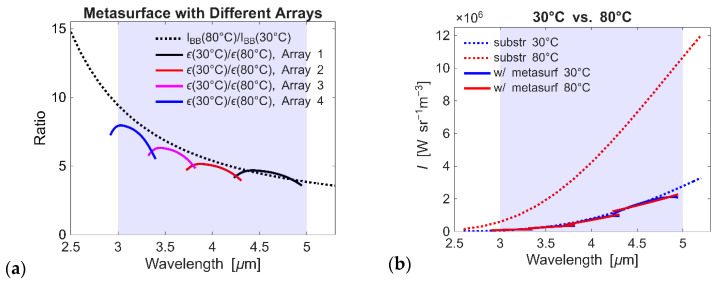
Compensation for multiple arrays: (**a**) Ratios I_BB_(80 °C)/I_BB_(30 °C) and ε(30 °C)/ε(80 °C) corresponding to the metasurface with four different 3 × 3 arrays. Each array provides compensation at the required wavelength. The set of four arrays is tailored to compensate for changes in blackbody radiation in the wavelength range λ ≈ 3–5 μm. (**b**) Radiance changes corresponding to temperatures of 30 °C and 80 °C for a bare silica substrate and a metasurface consisting of four arrays.

The library of pre-characterized antenna elements enables efficient metasurface design by allowing combinations of independently tuned resonators without the need to perform full-scale simulations of the entire structure. Designing and simulating the complete metasurface structure requires substantial computational resources due to the complexity and size of the parameter space, making direct full-scale modeling impractical. We design a metasurface structured from four separate arrays, where each array incorporates a 3 × 3 set of antennas arranged to achieve the desired response (Figure 7a). Array 1, the largest, is composed of 3 × 3 antennas with sides 1.915–2.150 μm in a lateral dimension of ~7.6 × 7.6 μm^2^; Array 2 has 9 antennas with sides 1.645–1.865 μm and of ~6.9 × 6.9 μm^2^; Array 3 has 9 antennas with sides 1.285–1.510 μm and of ~5.6 × 5.6 μm^2^; and Array 4, the smallest, has 9 antennas with sides 0.995–1.210 μm and of ~4.8 × 4.8 μm^2^. By combining the contributions of these arrays, the metasurface ensures continuous compensation throughout the full 3–5 μm spectral range (Figure 7b). Our analysis demonstrates that the resonance peak of each antenna element can be effectively tuned through geometric control and that these elements can be combined into metasurfaces with minimal inter-element interaction. This modular design approach allows for the construction of metasurfaces with tailored spectral responses using a limited set of pre-characterized building blocks. Most importantly, we demonstrated the ability to achieve the required 10-fold emissivity change, which necessitates sharp, high-contrast antenna resonances that cannot be realized with planar thin-film structures.

In metasurfaces designed to compensate emissivity at a single wavelength, introducing a central opening provides an efficient means of adjusting the relative strengths and spectral positions of the resonant modes supported by an individual antenna. As demonstrated in Figure 5g, the length of the hole side *h* governs the balance between the two key resonances: smaller openings enhance the longer-wavelength resonance, while larger openings selectively strengthen the shorter-wavelength mode. In addition, increasing the hole size shifts both resonances toward shorter wavelengths, providing a convenient degree of geometric tunability when only a single resonator must satisfy a targeted compensation condition. When operating with an array of antennas that contribute collectively, the design constraints differ. In this case, the overall emissivity compensation relies on the aggregate response of many elements rather than on the finely tuned response of an isolated resonator. Because the hole-induced modulation of modal strength is no longer required to isolate a specific wavelength, antennas without openings provide a more favorable and uniform resonance profile across the array. These solid antennas exhibit stronger and more spectrally consistent modes within the operational band. For this reason, a no-hole design is more advantageous for the multi-element compensation strategy pursued in Section 3.3. Further improvements are certainly possible. Hybrid arrays combining antennas with and without openings, or employing graded hole sizes across the surface, could offer additional control over resonance shaping, bandwidth tailoring, and spatially dependent compensation. Such composite designs, however, introduce a considerably larger optimization space and lie beyond the scope of the present study. Our objective here is to demonstrate the principle of multi-element emissivity compensation, for which the no-hole antennas represent the most effective and direct implementation. Finally, we note that we conducted similar analyses for other metals (silver and aluminum) and observed behavior very similar to that observed for gold, provided adjustments were made to certain geometric parameters, such as antenna dimensions and layer thicknesses. A broad parameter space is available for further optimization of metasurface performance.

## 4. Conclusions

In this work, we advance thermal coatings that play a crucial role in controlling the radiative properties of structures, devices, and systems, particularly in scenarios that require concealment or thermal regulation. We engineer a coating that enables thermal emission control using VO_2_, which undergoes insulator-to-metal phase transitions, resulting in significant variations in its optical properties with temperature. Our results demonstrate the potential to extend the operational spectral and temperature ranges for emission control by integrating phase-change materials into metasurfaces comprising arrays of metal–insulator–metal antennas. VO_2_ is employed to counteract radiance variations governed by blackbody emission in the spectral range of 3–5 μm. The emissivity of the engineered coating enhanced by the metasurface is strongly modulated by the phase transition of VO_2_ within each metasurface element. The proposed metasurface comprises antennas with various dimensions, and the lateral dimensions of the antenna enable control over the spectral position and strength of the resonance, compensating for changes caused by blackbody radiation. We examine the influence of antenna dimensions to minimize apparent temperature shifts. The resulting thermal coating effectively operates across the 3–5 μm spectral range and in the temperature range 30–80 °C.

Compared with existing compensation strategies, which typically rely on multilayer thin-film stacks [6], gradient-doped VO_2_ coatings [88], or external feedback and control circuits [89], the present metasurface offers a fundamentally different mechanism rooted in its patterned, multi-resonant architecture. Conventional unpatterned VO_2_ layers exhibit monotonic emissivity changes upon switching, requiring intricate thickness optimization or local control to achieve partial compensation. Similarly, multilayer dielectric–metal stacks can provide some spectral tailoring but lack the distributed set of resonant pathways required for the broadband response. In contrast, the proposed metasurface leverages many spatially distinct resonators whose thermal suppression collectively reshapes the absorption spectrum, yielding a wideband compensation effect without the need for external control, thermal biasing, or complex multilayer engineering. This qualitatively distinguishes the present approach as a passive, fabrication-compatible route to achieving broadband emissivity stabilization.

## Data Availability

The data that support the findings of this study are available from the corresponding author upon reasonable request.
